# Slow power-envelope associations among brain, heart, and gut signals during sleep: an exploratory multivariate transfer entropy study

**DOI:** 10.3389/fphys.2026.1924709

**Published:** 2026-10-20

**Authors:** Guojing Wang, Hongyun Liu, Shijing Wu, Xiaohua Yu, Weidong Wang

**Affiliations:** 1 Medical Innovation Research Division, Chinese PLA General Hospital, Beijing, China; 2 Key Laboratory of Biomedical Engineering and Translational Medicine, Ministry of Industry and Information Technology, Chinese PLA General Hospital, Beijing, China

**Keywords:** brain-heart-gut interaction, information flow, multimodal physiological signals, multivariate transfer entropy, sleep staging

## Abstract

Sleep and the brain-gut axis both involve the coordinated regulation of the central nervous system (CNS), autonomic nervous system (ANS), and enteric nervous system. Although the role of the brain-gut axis in sleep regulation is increasingly recognized, the dynamic information flow and its directionality among the brain, heart, and gut systems during sleep remain unclear as statistical associations. In this study, based on overnight sleep data from 24 healthy subjects, we simultaneously collected occipital electroencephalogram (EEG), electrocardiogram, and bowel sound signals, constructed a multimodal physiological signal analysis framework, and employed a multivariate transfer entropy method based on non-uniform embedding and *k*-nearest neighbor estimation to systematically quantify directed statistical associations at the level of slow power envelope co-modulations among the brain, heart, and gut systems within the framework of sleep staging. The results reveal a consistent pattern of directed statistical associations: information transfer from heart to brain, brain to gut, and heart to gut consistently exceeds that in the reverse directions across all sleep stages. Sleep-stage dynamics further dissociate within-system from inter-system information flow: internal information exchange within the brain and gut intensifies with sleep depth and weakens during REM, whereas inter-system pathways show divergent stage-dependent patterns: heart-to-brain flow increases from light to deep sleep, while heart-to-gut and brain-to-gut flow are suppressed during deep sleep. At the frequency-band level, the brain, heart, and gut each exhibit dynamically reorganized source–receiver roles across sleep stages, with specific frequency bands contributing differentially to each inter-system pathway depending on sleep stage. This study identifies exploratory statistical patterns of directional information flow among the brain, heart, and gut systems during sleep, providing hypothesis-generating observations for future studies on sleep and multi-organ interactions. These directed associations do not establish anatomical pathways, causal regulation, or clinical biomarkers, and should be regarded as exploratory.

## 1 Introduction

Sleep is a key physiological process for maintaining body homeostasis. Its regulation depends not only on the central nervous system (CNS) but also on peripheral signals, among which signals originating from the gut can communicate bidirectionally with the CNS via the brain-gut axis. Meanwhile, the heart, as a core observation window of autonomic nervous system (ANS) function, is a key hub in brain-gut dynamic interaction. Its activity is not only directionally regulated by brain rhythms but also forms reflex arcs with the gut through the vagus nerve, thus constituting the anatomical and physiological basis of the brain-heart-gut functional axis (Cryan et al., 2019; Richards et al., 2021). Existing studies have shown that brain-gut interaction is bidirectionally associated with sleep behavior (Sen et al., 2021; Wang et al., 2022d). Therefore, research on brain-heart-gut interaction during sleep has value for characterizing multi-organ statistical associations during sleep and generating hypotheses for future mechanistic and clinical studies.

Current research on sleep and brain-gut interaction mainly follows the core theoretical paradigm of the microbiota-gut-brain axis, focusing on analyzing the bidirectional regulatory relationship between sleep and gut microbiota and its underlying mechanisms (Wang et al., 2022e; Pan et al., 2025; Zhao et al., 2026). Although microbiota-gut-brain axis studies during sleep have revealed a close relationship between sleep and the gut at multiple levels, they still have significant limitations: most studies rely on static, discrete sampling methods such as fecal microbiota sequencing and blood index detection, resulting in a lack of understanding of the dynamic changes of the brain-gut axis during natural sleep and an inability to observe the real-time interaction among CNS, ANS, and ENS at a fine sleep staging scale. Therefore, conducting sleep-stage-specific dynamic studies of brain-gut interaction based on non-invasive continuous physiological signals is a core breakthrough for further understanding the mechanisms of sleep regulation and the pathophysiology of brain-gut disorders. Currently, relatively mature non-invasive continuous assessment methods have been developed for CNS and ANS functions. Among non-invasive CNS assessment methods, EEG-based electrophysiological technology is the gold standard for assessing cortical neural oscillations and the mainstream tool for sleep staging (Koizumi et al., 2022; Schoch et al., 2022). In ANS function assessment, HRV based on ECG signals has become the most commonly used non-invasive physiological indicator. Because it effectively characterizes autonomic nervous status, this indicator is used in brain-gut disorder research to assess overall and branch activities of the ANS (Mróz et al., 2022; Ali and Chen, 2023). However, current assessment of gut function still has significant technical limitations, becoming a bottleneck restricting in-depth research in this field. Gut motility assessment can effectively reflect the overall impact of neural regulation on gut function (Jeanne et al., 2023). However, traditional gut motility assessment methods mostly rely on invasive means such as manometry and biopsy (Ali et al., 2021; Shi et al., 2021), or imaging methods that are difficult to monitor continuously over long periods (Sclocco et al., 2022). The former has a high invasive burden, while the latter cannot meet the needs of long-term sleep monitoring. Currently available non-invasive gut motility assessment methods suitable for long-term observation mainly include abdominal surface electromyography (Orr et al., 1997) and bowel sounds (Goh et al., 2025). Abdominal surface EMG signals are susceptible to various interfering factors, limiting the accuracy of gut activity assessment based on them (Jeanne et al., 2023). In contrast, bowel sounds, as a non-invasive assessment tool, have been confirmed by multiple domestic and international studies to be effective in assessing gut motility and disease states such as functional bowel disorders related to brain-gut disturbances (Ficek et al., 2021; Wang et al., 2022a; Goh et al., 2025). Notably, the sleep state itself provides unique advantages for high-quality bowel sound acquisition: minimal body movement and low ambient background noise. Researchers have found that using bowel sounds to assess gut motility during sleep has significant advantages (Ficek et al., 2021), meeting the needs for non-invasive, long-term, low-interference monitoring. However, it should be noted that bowel sounds represent an indirect acoustic surrogate of gastrointestinal motility rather than a direct measure of intestinal contractile activity.

In recent years, the emergence of network physiology has provided a new paradigm for studying multi-system interactions (Ivanov, 2021). Traditional linear coupling analysis methods struggle to capture nonlinear couplings. Although nonlinear coupling methods based on mutual information can effectively characterize nonlinear interactions between systems (e.g., our previous use of the maximal information coefficient method to quantitatively assess brain-heart-gut coupling during sleep), such methods still cannot quantify the directionality and timing of information flow (Platiša et al., 2023; Tian et al., 2024). To address this, researchers have proposed causal inference approaches, whose core is to identify directional influences between variables. Wiener’s prediction criterion laid the philosophical foundation for this: if the history of process X effectively reduces the uncertainty in predicting the future of process Y, then X has a causal influence on Y (Wiener, 1956). Granger placed this criterion within a linear autoregressive framework, forming Granger causality (Granger, 1969). However, its linear assumption has fundamental limitations in characterizing the nonlinear dynamics of complex physiological systems (Platiša et al., 2023). To overcome this limitation, Schreiber placed Wiener’s criterion within a more general information theory framework and proposed transfer entropy (TE) (Schreiber, 2000). TE is a model-free information-theoretic method that effectively quantifies directional information transfer between time series and has been widely used in studies of brain functional networks and brain-heart coupling (Harmah et al., 2019; Yang et al., 2021). However, traditional bivariate TE is susceptible to common driving and cascading effects in multivariate systems, making it difficult to distinguish direct from indirect causal associations (Xi et al., 2021). To accurately identify direct causal relationships, conditionalization is necessary to remove indirect influences (Ekhlasi et al., 2023). Multivariate transfer entropy (MuTE), by conditioning on the states of other systems, can precisely identify direct information flow in multivariate systems (Harmah et al., 2019; Yang et al., 2021; Wang et al., 2022b), providing a methodological basis for analyzing the complex interactions among the brain, heart, and gut systems.

This study takes sleep staging as the analytical framework, and based on synchronously collected multimodal physiological signals (EEG, ECG, and bowel sounds), systematically investigates the envelope-level directional information flow among the brain, heart, and gut systems under different sleep stages using the MuTE method, revealing the dynamic patterns of slow power-envelope statistical associations across sleep stages, as an exploratory contribution that may inform future mechanistic and clinical investigations.

## 2 Materials and methods

### 2.1 Subjects

A total of 32 individuals were assessed for eligibility. Of these, 8 were excluded: 5 did not meet the good sleeper criteria, 0 declined to participate, and 3 were excluded due to incomplete data. The remaining 24 healthy subjects met all inclusion criteria and completed the overnight PSG recording. No additional exclusions were made after data quality assessment. A participant flow diagram is provided in **Supplementary Figure S1**. and the demographic characteristics and sleep data of these 24 subjects are summarized in **Table 1**.

**Table 1:** Basic information of participants.

Characteristic	Mean ± SD	Range (Min-Max)
Male/female	12/12	–
Age(y)	29 ± 4	24-38
Body Mass Index	22.89 ± 4.08	18.37-28.87
Fraction of LS	0.58 ± 0.08	0.47-0.73
Fraction of DS	0.22 ± 0.07	0.09-0.35
Fraction of REM	0.20 ± 0.06	0.13-0.28

All 24 subjects met the criteria for good sleepers, adapted from (Ramlee et al., 2017) and consistent with AASM guidelines for normal sleep: (1) no history of insomnia or other sleep disorders; (2) sleep latency < 15 minutes; (3) average total sleep time approximately 7–9 hours (consistent with the AASM/SRS consensus recommendation for healthy adults); and (4) number of awakenings after sleep onset < 2. These criteria were verified through a combination of subjective self-report, PSG-based objective measurement, and video monitoring. Subjects not meeting any of these criteria were excluded. In addition, subjects with a history of cardiovascular, neurological, or gastrointestinal diseases were excluded. PSG was used to simultaneously collect parameters such as oronasal airflow and thoracic/abdominal respiratory movements. Sleep events were annotated by professional technicians to exclude those with obstructive sleep apnea (OSA). Consistent with the American Academy of Sleep Medicine (AASM) clinical practice guidelines, OSA was defined as an apnea-hypopnea index (AHI) ≥ 5 events/h; Subjects with AHI ≥ 5 were excluded from the study.

Before the experiment, subjects were asked to eat a light meal or fast for dinner and to refrain from alcohol, caffeine-containing beverages (coffee, tea, energy drinks), and sleep-affecting medications for at least 24 h prior to the PSG recording. Compliance was verified through self-report questionnaires administered at the time of laboratory arrival; no subject reported recent use of sleep medications or consumption of alcohol/caffeine within the 24 h window.

This study was approved by the Medical Ethics Committee of the First Medical Center of the People’s Liberation Army General Hospital (Approval No. S2022-341-01), and all subjects signed written informed consent.

### 2.2 Data acquisition

A key prerequisite for conducting research on brain-heart-gut interactions during sleep is the high-quality synchronous acquisition of EEG, ECG, and bowel sound signals. To this end, this study used a PSG system to simultaneously acquire EEG and ECG signals, and sleep staging was manually annotated by professionals based on PSG recordings; bowel sound signals were collected separately using a self-developed bowel sound recorder.

The PSG system used was the N7000 model manufactured by Embla Systems (USA), which can simultaneously acquire and store multi-dimensional physiological signals such as sleep EEG, ECG, electrooculography, electromyography, respiration, snoring, body position changes, and blood oxygen saturation. For the objectives of this study, the system provided high-quality sleep EEG, ECG signals, and standardized sleep staging data. The specific acquisition configuration was as follows:

1. Sleep EEG signal acquisition: A 6-lead EEG recording mode was used, with electrodes placed strictly according to the international 10–20 system standard at positions F3, F4, C3, C4, O1, and O2.
  Reference electrodes were placed on the bilateral mastoids (A1, A2). The signal sampling rate was set to 500 Hz.
2. Sleep ECG signal acquisition: A standard three-lead limb lead mode was used, with electrodes placed under the right clavicle, left clavicle, and left lower abdomen. The sampling rate was also set to 500 Hz to ensure the morphological integrity and high recognizability of key ECG waveforms such as QRS complexes, P waves, and T waves, providing high-quality signals for subsequent heart rate variability analysis and ANS function assessment.
3. Manual sleep staging annotation: Sleep staging strictly followed the standardized scoring manual published by the American Academy of Sleep Medicine (AASM) in 2007 and the updated 2017 version (Berry et al., 2017). This standard divide overnight sleep into five core stages: Wake, NREM (N1, N2, N3), and REM, where N1 and N2 are combined as light sleep (LS) and N3 as deep sleep (DS). All PSG recordings were manually annotated frame by frame at 30-second epochs by professionally trained and certified sleep technicians. This study focused on directed statistical associations among systems during sleep, so only the sleep stages LS, DS, and REM were included for analysis (Yang et al., 2021; Wang et al., 2022b). Finally, comparative analysis of MuTE was performed under three sleep states—LS, DS, and REM—allowing reflection of interaction characteristics during sleep at a global level while further distinguishing heterogeneity across different sleep stages.

Bowel sound signals were collected using a self-developed bowel sound recorder (Wang et al., 2023), which was equipped with two SiSonic series patch-type MEMS microphones from Knowles Corporation. These microphones feature a high signal-to-noise ratio of 69 dB, an ultra-wideband flat frequency response of 10-10000 Hz, and tight sensitivity matching of ±3 dB, effectively covering the main frequency band of bowel sounds (100-1000 Hz) (Wang et al., 2022a). One microphone was used to collect bowel sound signals, and the other simultaneously collected ambient noise to provide a reference for subsequent adaptive noise reduction, with a sampling rate of 8000 Hz. To reduce heart sound interference, the bowel sound recorder was placed on the surface of the right lower abdomen.

Since the PSG system and the bowel sound recorder were two completely independent devices, specific synchronization methods were necessary to achieve temporal alignment of the signals. Therefore, we adopted a two-end tapping strategy of “start-alignment + end-validation”. At the beginning of each night’s recording, the sensors of the two devices were tapped together to generate a transient synchronization marker for initial alignment. Specifically, the leg electromyography sensor of the PSG system and the pickup port of the bowel sound recorder were tapped together, causing both independent devices to generate simultaneous markers. At the end of the recording, a second tapping was performed to generate an additional set of markers, which were used to correct the sampling rate of the bowel sound data and eliminate overnight clock drift.

### 2.3 Data processing

The main purpose of data processing was to improve signal quality and extract frequency power sequences of sleep EEG, ECG, and bowel sound signals. Considering that body movements during sleep can easily mask bowel sound and EEG signals (Lyu et al., 2023), this study first excluded power sequence segments containing body rollovers to effectively control the impact of motion artifacts. Specifically, rollover segments were detected using information from the Activity channel obtained by the accelerometer in the PSG system, and by manually setting the threshold to 0.2, rollover segments were accurately identified and removed (Kuo et al., 2016).

#### 2.3.1 Sleep EEG

For the analysis of sleep EEG signals, although this study acquired EEG signals from six channels (F3, F4, C3, C4, O1, O2), considering that the core objective was to investigate the overall coupling laws of the brain-heart-gut system under different sleep stages rather than functional localization of brain regions, the O2 lead was selected as the representative channel for EEG in subsequent feature extraction and analysis. This choice was mainly based on the following considerations: First, in system-level brain-body coupling studies, selecting a single representative channel helps reduce the dimensionality of multivariate analysis and avoid information redundancy while preserving the main characteristics of overall CNS activity; similar strategies have been widely adopted in existing brain-heart coupling studies (Faes et al., 2015; Yang et al., 2021). Second, according to the AASM guidelines, occipital alpha waves are a key feature distinguishing Wake/REM from other NREM stages, and the occipital region also responds well to slow-wave activities such as delta waves, meeting the basic requirements for sleep staging determination (Rodenbeck et al., 2015; Jorgensen et al., 2020). Third, the O2 lead, located in the occipital region, is relatively less affected by electrooculography and electromyography artifacts, providing stable signal quality, which is an important factor ensuring the reliability of subsequent feature extraction and coupling analysis. Furthermore, existing studies have shown that signals related to CNS and cardiorespiratory coupling exhibit significant occipital dominance, suggesting that the occipital region may be a key brain area for receiving and processing information interaction in the CNS-cardiorespiratory network system (Yang et al., 2021). Based on the above reasons, this study uniformly used the O2 lead EEG signal as the representation of CNS activity in subsequent analyses. We acknowledge that O2 activity does not represent whole-brain information flow; therefore, all subsequent references to “brain” or “CNS” activity in this study refer specifically to occipital EEG activity recorded from the O2 lead, unless otherwise specified.

For EEG signal processing, a fourth-order Butterworth bandpass filter (passband 0.5-40 Hz, forward and reverse filtering using the *filtfilt* function) was first applied to remove low-frequency drift and high-frequency interference (Wang et al., 2022b; Lyu et al., 2023). Prior to spectral analysis, all 4-second epochs underwent visual inspection: epochs in which the EOG channel amplitude exceeded 100 μV were regarded as ocular artefacts and removed via wavelet transform; epochs showing obvious high-frequency activity in the EMG channel were considered muscular artefacts and were suppressed using the Multivariate EMD and CCA method for removing muscle artefacts from few-channel EEG recordings (Chen et al., 2017). After preprocessing, a sliding window of length 4 seconds with 1-second step was used to calculate the power spectral density, yielding a power time series with a resolution of 1 Hz (Valenza et al., 2016). This operation converts the raw oscillatory signals into slow amplitude co-modulations, meaning that our subsequent MuTE analysis quantifies information flow at the envelope level rather than at the level of the original carrier oscillations. Specifically, Welch’s periodogram method was used to calculate the relative power spectral values for four frequency bands: delta (0.5-4.0 Hz), theta (4.0-8.0 Hz), alpha (8.0-13.0 Hz), and beta (13.0-30.0 Hz).

#### 2.3.2 Sleep ECG

To meet the research requirements for assessing ANS regulation during sleep, it was necessary to convert ECG signals into RR interval (RRI) sequences for heart rate variability (HRV) analysis (Cheng et al., 2023). Therefore, R waves needed to be extracted from the raw ECG signal to construct the RRI sequence, followed by power spectral density estimation. The specific procedure was as follows: The raw ECG signal was filtered using a fourth-order Butterworth bandpass filter (0.5-40 Hz) (Wang et al., 2022b). Then, the Haar wavelet transform combined with an adaptive threshold method was used to detect QRS complexes, and the point with the maximum amplitude in each complex was located as the R-wave peak. After obtaining R-wave positions, the mean and standard deviation of the RRI sequence were calculated using a 21-point moving window. When the normalized deviation of a data point exceeded a preset threshold (set to 4), that point was replaced with the window mean (Pilia et al., 2021; Lyu et al., 2023). Next, detrending was performed on the RRI sequence using db12 wavelet transform, followed by uniform interpolation and resampling at 8 Hz. For power spectral density estimation, an autoregressive (AR) model based on the Burg algorithm was used. The AR model order was determined using the final prediction error (FPE) criterion, with the search range set from 8 to 25; the order was finally selected as 16. Combined with a sliding window of length 120 seconds and 1-second step, to calculate the relative power values for the low-frequency (LF, 0.04-0.15 Hz) and high-frequency (HF, 0.15-0.40 Hz) bands, ultimately generating an HRV power time series with a resolution of 1 Hz, which likewise represents the slow power envelope of cardiac autonomic outflows rather than the raw R-R interval dynamics. In addition, within the LF and HF bands, the spectral peak frequency was defined as the local maximum of the AR power spectral density; the power-weighted centroid frequency for each band was also computed to reflect possible shifts in the peak within the band. These spectral components are referred to as HRV spectral measures rather than direct markers of specific autonomic branches.

#### 2.3.3 Sleep bowel sounds

For processing sleep bowel sound signals, this paper adopted a three-stage preprocessing procedure including bandpass filtering, adaptive filtering, and wavelet threshold denoising (Wang et al., 2023). First, the bandpass filter used was a fourth-order Butterworth filter with cutoff frequencies of 100 Hz and 1000 Hz (Wang et al., 2022a, 2023). On this basis, a normalized least mean squares (NLMS) algorithm was introduced for adaptive noise reduction based on the dual-channel configuration, with a filter order of 64 and a step size of 0.001 (Al-Naggar and Al-Udyni, 2018; Wang et al., 2023). Subsequently, sym6 wavelet basis was used to perform 6-layer wavelet decomposition on the signal, and thresholds for each layer were calculated according to the Birge-Massart strategy to complete wavelet threshold denoising (Sidhik, 2015; Wang et al., 2023). After completing the above denoising processing, the Welch periodogram method was applied to estimate the power spectral density of the signal using a sliding window of length 4 seconds and 1-second step. The relative power spectral values were calculated for five frequency bands: BS1 (100-200 Hz), BS2 (200-300 Hz), BS3 (300-400 Hz), BS4 (400-500 Hz), and BS5 (500-1000 Hz), thereby obtaining the power time series corresponding to each frequency band. Relative power for each band was calculated as the ratio of the band-specific power to the total power across the entire 100-1000 Hz range. The frequency band boundaries were predefined based on empirical spectral distributions reported in the bowel sound literature: (1) normal bowel sounds are primarily distributed within 100–1000 Hz (Wang et al., 2023; Geng et al., 2026); (2) healthy adult bowel sounds exhibit a peak frequency around 340 Hz with main energy concentrated in 200–550 Hz; and (3) colonic sounds tend to have lower frequencies (~100 Hz) whereas gastric/small intestinal signals exhibit higher frequencies (~300 Hz) (Sood et al., 2026). However, these boundaries are empirically derived and have not been independently validated against concurrent manometric or imaging measurements. To minimize the confounding effects of technical and anatomical factors on the acoustic spectrum—including sensor characteristics, abdominal wall transmission, and sensor placement—we focused on within-subject comparisons across sleep stages, where these factors remain invariant throughout the overnight recording. For potential time-varying confounders, we positioned the recorder on the right lower abdomen to reduce cardiac acoustic interference, applied a 100 Hz high-pass filter, and used a dual-channel adaptive noise cancellation algorithm. Body movement segments were excluded to control for positional changes and motion artifacts. As with the EEG and HRV signals, these power time series represent the slow energy envelopes of bowel sound activity, not the raw acoustic waveforms.

### 2.4 Multivariate transfer entropy

MuTE is an information-theoretic, model-free approach for quantifying directed information flow among nodes in a multivariate system. In this study, MuTE was employed to construct the brain-heart-gut information flow network, aiming to overcome the limitations of conventional bivariate transfer entropy (TE) which is susceptible to cascade effects and common driver effects in multi-system interactions, thereby enabling accurate identification of direct causal relationships (Montalto et al., 2014).

Consider the composite system *S*= {*X*, *Y*, *Z*} consisting of the brain system *X*, the cardiac system *Y*, and the gut system *Z*. Here, *X* comprises four EEG frequency bands (delta, theta, alpha, beta), *Y* comprises two HRV frequency bands (LF, HF), and *Z* comprises five bowel sound frequency bands (BS1–BS5). The state of each node is represented by the corresponding relative power time series. To retain sufficient statistical samples while capturing rapid physiological variations, a non-overlapping sliding window of 5 minutes (300 data points at a sampling rate of 1 Hz) was used for analysis, separately for DS, LS, and REM sleep. The MuTE calculation proceeds as follows.

Step 1: Construction of the candidate matrix

For a given window, let the target node be *T* (e.g., an EEG frequency band). The past values of all nodes in system *S* (including *T* itself) are expanded according to a preset maximum time delay *μ* to form the candidate matrix (CM). Specifically, for target node *T*, the CM contains the following vectors:


$$\text{CM}=\{T_{1:m-1},\cdots ,T_{1:m-\mu },X_{1:m-\mu }^{1},\cdots ,X_{1:m-\mu }^{n_{X}},Y_{1:m-\mu }^{1},\cdots ,Y_{1:m-\mu }^{n_{Y}},Z_{1:m-\mu }^{1},\cdots ,Z_{1:m-\mu }^{n_{Z}}\}$$

where *m*=300 is the window length, *μ*=10 s (i.e., the maximum information transfer delay), and *n_X_*, *n_Y_*, *n_Z_* are the numbers of nodes in each subsystem. This candidate matrix encompasses all lagged variables that might be predictive of the current state *T_n_* of the target node.

Step 2: Non-uniform embedding

To avoid the high-dimensional sparsity problem caused by uniform embedding, an iterative conditional mutual information maximization strategy is used to select the most relevant lagged variables (Vlachos and Kugiumtzis, 2010). Let *W* be the set of already selected variables (initially empty). In each iteration, for each candidate variable *v* in the CM, the conditional mutual information with the target variable *T_n_* is computed as:


$$I(T_{n};v|W)=H(T_{n}|W)-H(T_{n}|W,v)$$

where the (conditional) entropies are estimated using a *k*-nearest neighbor estimator (see Step 3). The candidate *v*^∗^ that maximizes $I(T_{n};v|W)$ is selected. Its statistical significance is assessed by a randomization test (block shuffling). If *v*^∗^ meets the significance level (*α*=0.01, 500 surrogates), it is added to *W* and removed from the CM, and the iteration continues; otherwise, the selection process stops. The final *W* constitutes the optimal embedding vector $V=[V^{X},V^{Y},V^{Z}]$, where *V^X^*, *V^Y^*, *V^Z^* represent the lagged variables of the brain, heart, and gut systems that significantly contribute to predicting *T_n_*.

Step 3: *k*-nearest neighbor entropy estimation

To accommodate the non-Gaussian, nonlinear characteristics of physiological signals, the joint *k*-nearest neighbor method proposed by Kraskov et al. is adopted to estimate conditional entropy (Kraskov et al., 2004), minimizing estimation bias across spaces of different dimensions. For any two random vectors *A* and *B*, the conditional entropy is estimated as:


H(A|B)=−〈logp(A|B)〉=ψ(k)+〈logvol(B)−logvol(A,B)〉+ψ(N)

where *ψ*(·) is the digamma function, *N* is the number of samples, and vol(·) denotes the volume of the hypersphere containing the *k*-th nearest neighbor in the respective space. In this study, *k* =10 (Wang et al., 2022c). This estimator uses a unified neighborhood scale determined in the highest-dimensional joint space and then performs range searches in all lower-dimensional projected subspaces, achieving an unbiased estimation of the multiple entropy terms.

Step 4: Calculation of multivariate transfer entropy

After obtaining the optimal embedding vector *V*, the multivariate transfer entropy from the source system *X* to the target node *T*, conditioned on the remaining system *Z*, is defined as:


$$TE_{X\to T}|Z=H(T_{n}|V^{T},V^{Z})-H(T_{n}|V^{T},V^{X},V^{Z})$$

where *V^T^* are the lagged variables belonging to the target node’s own history in *V*, and *V^Z^* are the lagged variables of the conditioning system (here, the gut system). This quantity measures the additional information provided by the history of the source system about the current state of the target system, beyond that already provided by the target’s own history and the histories of the other systems. If at least one lagged variable from the source system *X* is selected during the non-uniform embedding procedure, then TE>0 and it is considered statistically significant; otherwise, TE=0.

Step 5: Network construction and statistical comparison

For every combination of (source system, target system, conditioning system) and every combination of frequency-band nodes, Steps 1-4 are repeated, finally constructing a directed brain-heart-gut information flow network with frequency-band resolution. To compare information transfer strengths across different sleep stages and to perform directional and band-specific comparisons, we employed a Linear Mixed Model (LMM). Specifically, the TE value from each time window was used as the dependent variable, sleep stage (light sleep, deep sleep, REM) as a fixed effect, and participant ID as a random intercept to account for repeated measurements from the same individual. By including the participant random effect, the LMM appropriately partitions the total variance into between-participant and within-participant components, thereby correcting for the non-independence of multiple windows from the same participant and avoiding bias due to unequal numbers of windows across stages. For multiple comparisons, we applied the Benjamini-Hochberg procedure to control the false discovery rate (FDR). Multiple-testing correction was organized into four pre-specified, non-overlapping families, each corrected jointly with the Benjamini-Hochberg procedure at q < 0.05: the connection-level main effect of sleep stage for each of the 110 directed connections; directional (input vs. output) comparisons (42 tests, pooled across figures); pairwise between-band contrasts (102 tests); and pairwise between-stage contrasts (150 tests). Every *p*-value belonged to exactly one family and was corrected exactly once; post-hoc pairwise stage contrasts following a significant main-effect connection were additionally Bonferroni-corrected (multiplier = 3) (**Supplementary Table S1**). Adjusted p-values (q < 0.05) were considered statistically significant. Data are presented as window-level mean ± standard error across windows (the same analytical unit used in the linear mixed model).

## 3 Results

This study presents the directed information flow patterns of the brain-heart-gut network across different sleep stages from three perspectives: macro-level characteristics of overall network information transfer, dynamic roles of intra-system band interactions, and inter-system band-specific pathways. Regarding the distribution of valid windows, the total numbers of valid 5-min windows for each sleep stage were as follows: LS 1,097 windows (mean 45 ± 12 windows per participant), DS) 403 windows (mean 16 ± 6 windows per participant), and REM sleep 388 windows (mean 16 ± 6 windows per participant). After data cleaning, the overnight sleep data included 24 participants, with the following valid window counts per stage: LS 1,069 windows (mean 44 ± 12 windows per participant), deep sleep (DS) 398 windows (mean 16 ± 6 windows per participant), and REM sleep 376 windows (mean 15 ± 6 windows per participant).

### 3.1 Macroscopic characteristics of global network information transfer: directional asymmetry in statistical associations

To assess the global information flow characteristics of the brain-heart-gut network, this study calculated and analyzed the MuTE matrices for each group under different sleep stages, as shown in **Figure 1**. The matrix is intuitively presented as a heatmap, where each element quantitatively represents the directional information transfer strength from the specific driving node (vertical axis) to the target node (horizontal axis), with color depth reflecting the transfer strength. As displayed in **Figures 1A–C**, compared to the reverse pathways, the heatmap colors for the heart-to-brain, heart-to-gut, and brain-to-gut directions are warmer, indicating that their information transfer strengths are generally higher than those of the corresponding reverse pathways. **Figure 1D** further corroborates this directional bias by presenting statistical analyses of inter-system information transfer strengths across different sleep stages. This feature is stable across different sleep stages, jointly outlining a consistent pattern of directed statistical associations: TE estimates in the heart-to-brain, brain-to-gut, and heart-to-gut directions were higher than in the reverse directions across all sleep stages.

**Figure 1:**
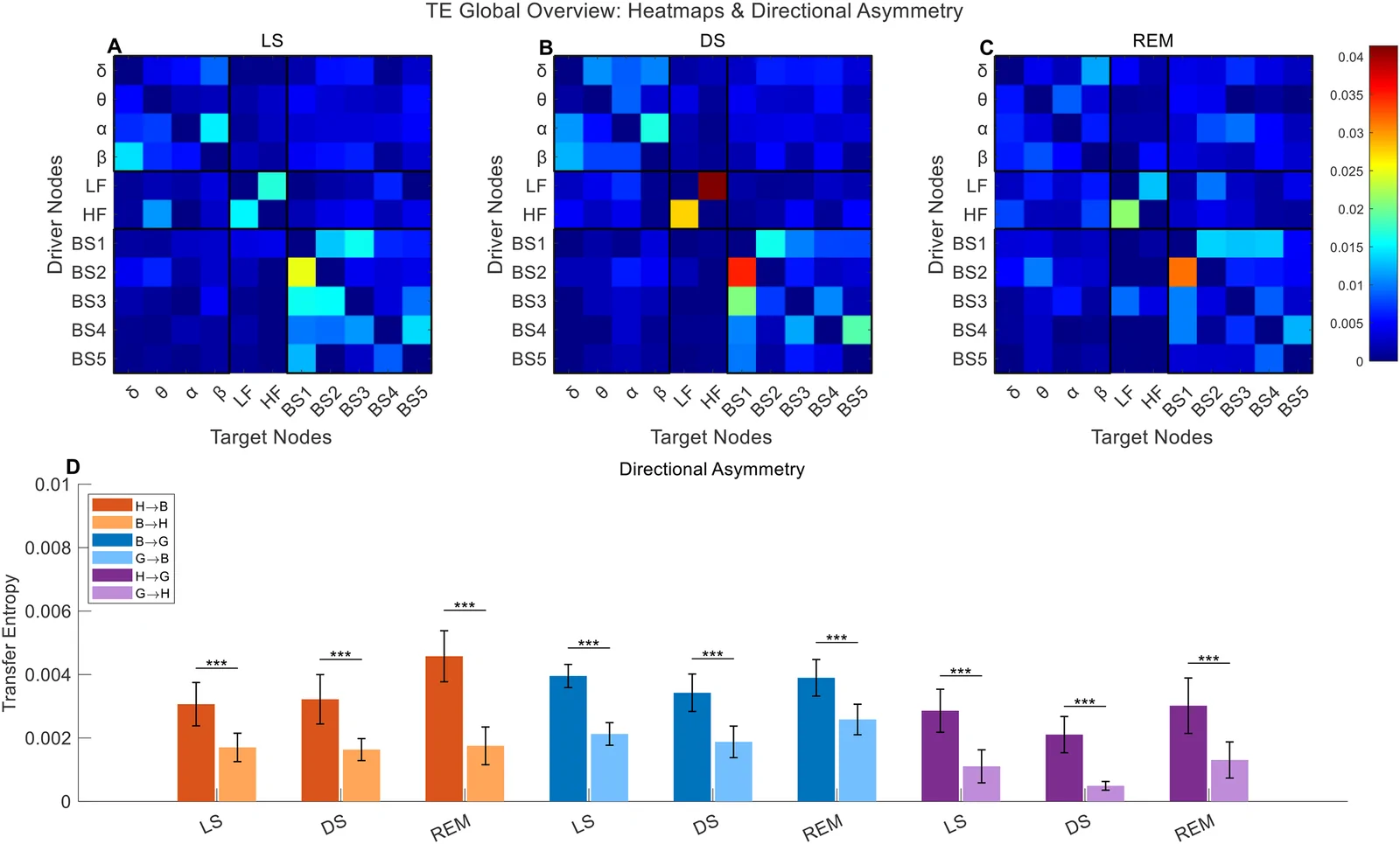
Directed information transfer strength matrix of the brain-heart-gut network under different sleep stages. **(A)** LS, **(B)** DS, **(C)** REM, and **(D)** directional asymmetry. H, heart; B, brain; G, gut.*** indicates *P*<0.001. Values are window-level transfer entropy (mean ± SE across windows), matching the unit of analysis of the linear mixed model.

Furthermore, information transfer both within and between the brain, heart, and gut systems also varies across sleep stages. As shown in **Figures 2A–C**, it can be seen that as sleep deepens from LS to DS, the information transfer strength within the brain, and gut systems gradually increases. whereas changes in the internal information intensity within the heart show no statistically significant difference. However, during REM sleep, this enhanced internal information transfer generally weakens.

**Figure 2:**
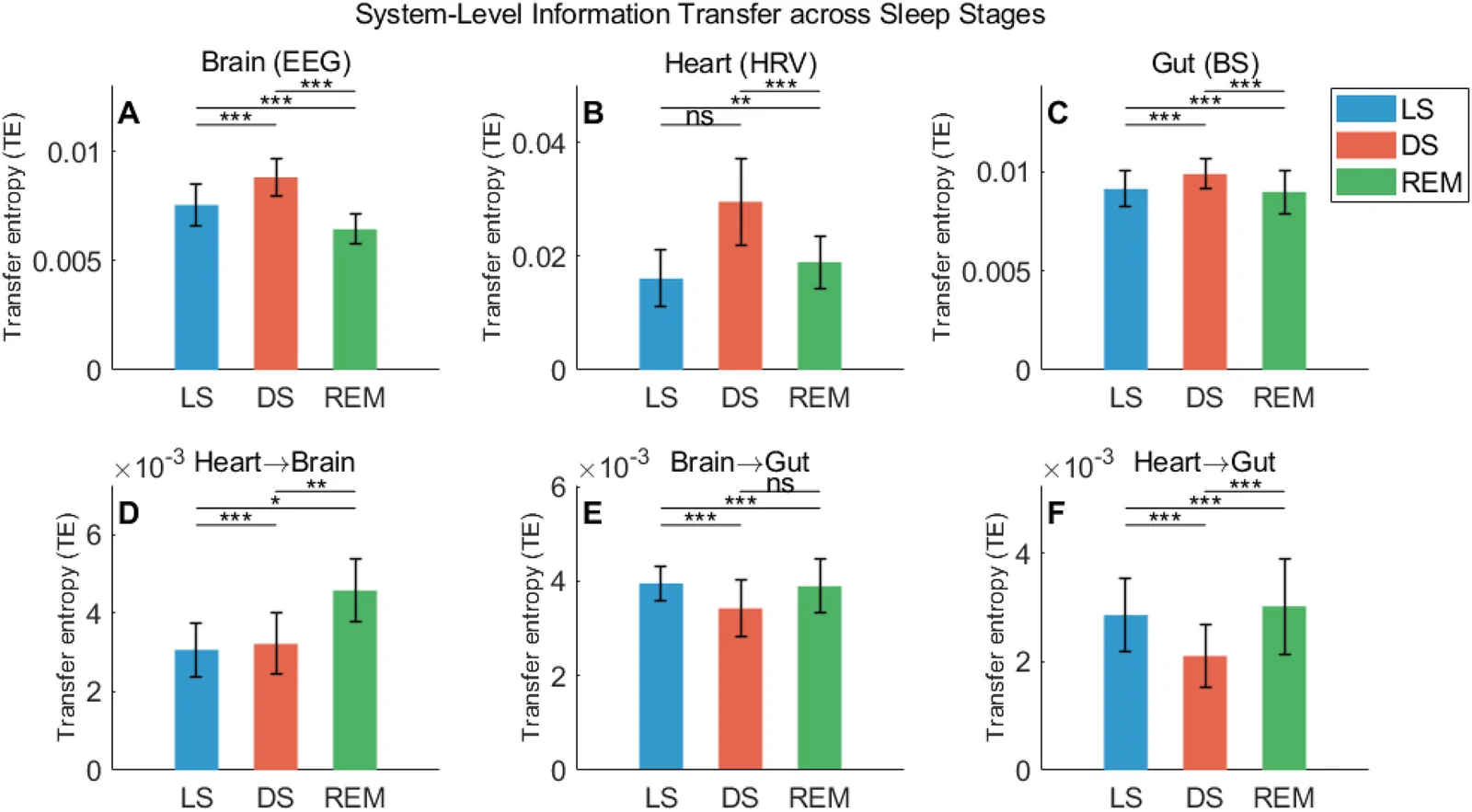
Changes in information transfer strength within and between the brain, heart, and gut systems under different sleep stages. **(A)** Within-brain, **(B)** within-heart, **(C)** within-gut, **(D)** heart-to-brain, **(E)** heart-to-gut, and **(F)** brain-to-gut. ns *P*>0.05; *P<0.05; **P<0.01; ***P<0.001. Values are window-level transfer entropy (mean ± SE across windows), matching the unit of analysis of the linear mixed model.

In contrast, the response of inter-system information transfer to sleep depth follows a different pattern, as shown in **Figures 2D–F**. Specifically, the information flow strengths along the heart-to-gut and brain-to-gut pathways decrease during deep sleep. Among these, the heart-to-brain and heart-to-gut pathways show a recovery during REM sleep, whereas the brain-to-gut pathway does not exhibit a significant rebound.

### 3.2 Dynamic role analysis of frequency band interactions within systems

To deeply reveal the information transfer within each system, this study further focused on the frequency band level. We quantified the functional role of each frequency band as a source (outgoing information) and a receiver (incoming information) and examined the dynamic shifts of these roles across sleep stages.

Within the CNS, different EEG frequency bands exhibit clear differentiation in their envelope-level information flow roles and dynamic reorganization as sleep deepens, as shown in **Figure 3A**. During LS, the alpha band serves as the primary statistical information source, while the delta and theta bands serve as the primary statistical information receivers; no significant difference is observed between the incoming and outgoing strengths of the beta band. Upon entering DS, the delta and alpha bands switch to information sources, whereas the theta band becomes information receivers. During REM sleep, no significant differences between incoming and outgoing strengths are found for any of the bands.

**Figure 3:**
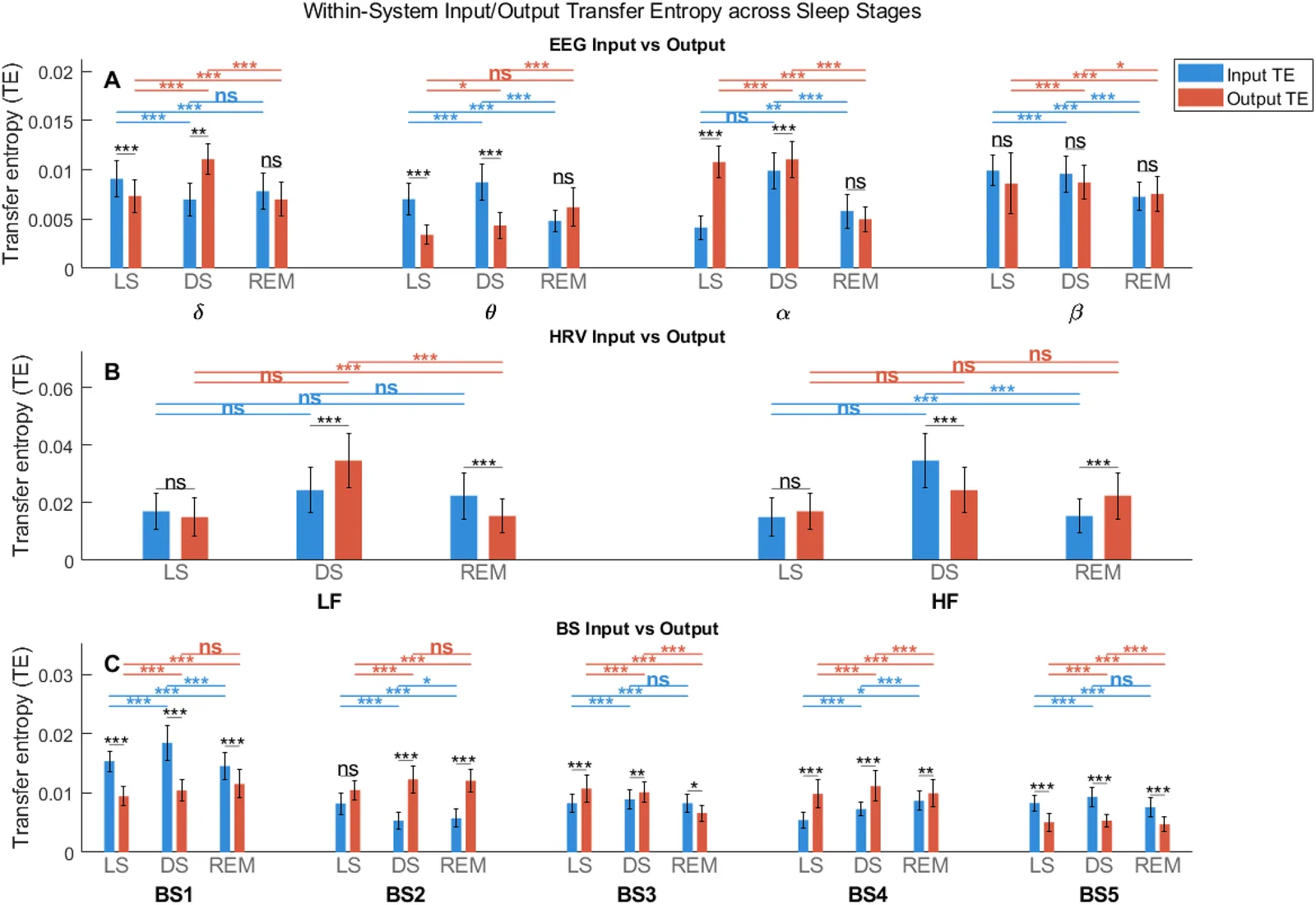
Changes in input–output strength across different frequency bands within distinct systems as a function of sleep stage. **(A)** EEG input vs. output, **(B)** HRV input vs. output, and **(C)** bowel sound (BS) input vs. output. ns *P*>0.05; *P<0.05; **P<0.01; ***P<0.001. Values are window-level transfer entropy (mean ± SE across windows), matching the unit of analysis of the linear mixed model.

Regarding the changes in information transfer strength across sleep stages, as shown in **Figure 3A**: in terms of incoming information strength, except for the alpha band, which shows no statistically significant difference, the delta and beta bands show a decreased strength during deep sleep, while the other bands reach their peak during deep sleep. In terms of outgoing information strength, all bands show an increasing trend from light sleep to deep sleep, and all bands except theta show a decline during REM sleep.

Within the ANS, the LF and HF bands of RRI form a stable and clear statistical driver-recipient pair at the envelope level, as shown in **Figure 3B**. During LS, there is no statistically significant difference between the incoming and outgoing information strengths of the LF and HF bands. Upon entering DS, LF shifts to being a statistical information source, while HF becomes a statistical information receiver. During REM sleep, the statistical roles of the two bands reverse again, with LF turning into a receiver and HF into a source. Regarding changes in information transfer strength with sleep stage, whether as information sources or receivers, the information transfer strengths of the LF and HF bands show no statistically significant changes from LS to DS, but from DS to REM, the intensity of information output from LF decreased, and the intensity of information input from HF also decreased.

Bowel sound power envelopes also show corresponding specificity in their envelope-level information flow roles, as shown in **Figure 3C**. During NREM sleep, the BS2, BS3, and BS4 bands tend to act as statistical information sources, while the BS1 and BS5 bands serve primarily as statistical information receivers. Except for the BS2 band, which shows no statistically significant difference between incoming and outgoing information strengths during REM sleep, and the BS3 band, whose information transfer role changes during REM sleep, the roles of the remaining bands remain generally unchanged across sleep stages. Additionally, the changes in information transfer strength of bowel sound frequency bands with sleep stage are specific. Compared with LS, during DS: the incoming information strengths of the BS2 band decrease, while its outgoing information strengths increase; the incoming strength of the BS3 band as a receiver increases, while its outgoing strength as a source decreases; and both incoming and outgoing information strengths of the BS1, BS4 and BS5 bands increase. These frequency-selective changes suggest that the intestinal physiological activities corresponding to different frequency bands may exhibit differentiated information processing patterns at the envelope level. However, it should be noted that this interpretation is based on information-theoretic measures of acoustic signals and does not directly map to specific intestinal contraction patterns.

### 3.3 Frequency-band-specific pathway analysis of inter-system information transfer

Based on the finding of dominant information flow directions in the global network, this study further performed frequency band-resolved analysis of information transfer among the three systems to reveal the frequency-level structure of the directed associations.

In the statistically dominant heart-to-brain information transfer pathway, which exhibits statistically stronger flow in the heart-to-brain direction, the frequency band contribution of the ANS as the driving end depends on sleep stage, as shown in **Figure 4**. During LS, the outgoing information strength of the HF band is higher than that of the LF band; but during DS and REM, there is no statistically significant difference between LF and HF. On the EEG receiving side, during LS, the theta band receives the highest information flow strength; during DS, the delta, theta, and alpha bands receive higher information strengths than the beta band; and during REM, the beta band receives the highest strength. With regard to changes across sleep stages, on the output side of the heart-to-brain pathway, the outgoing strength of the LF band shows no statistically significant difference from LS to DS, whereas the HF band reaches its lowest level during DS. On the input side, the incoming strength of the delta and alpha bands reach their highest from LS to DS, while other bands reach to their lowest during DS.

**Figure 4:**
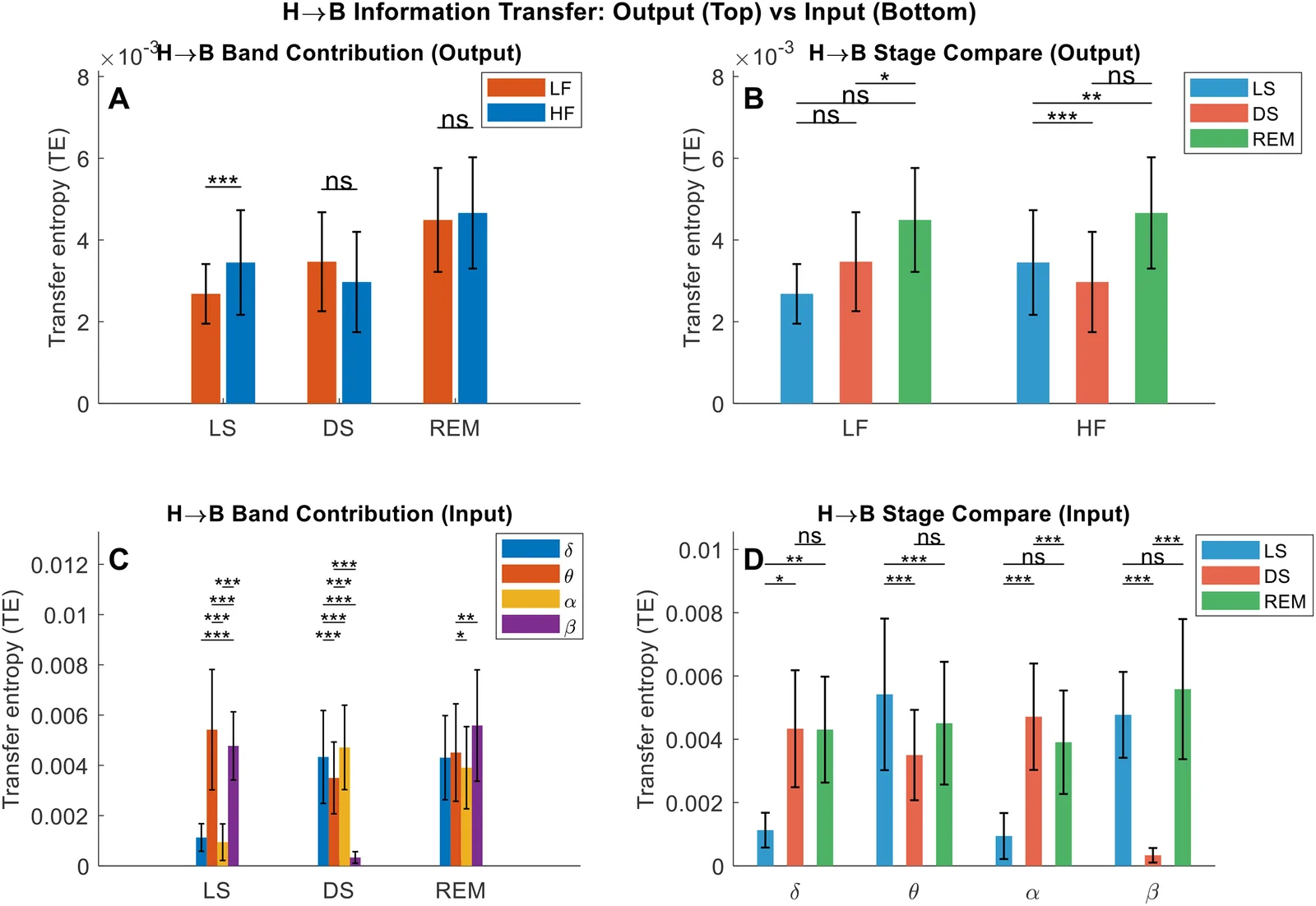
Changes in the strength of information flow in the input and output directions across different frequency bands in the heart-to-brain direction as a function of sleep stage. **(A)** Heart-to-brain output band contribution, **(B)** heart-to-brain output stage comparison, **(C)** heart-to-brain input band contribution, and **(D)** heart-to-brain input stage comparison. ns *P* > 0.05, *P < 0.05, **P < 0.01, ***P < 0.001. Values are window-level transfer entropy (mean ± SE across windows), matching the unit of analysis of the linear mixed model.

In the brain-to-gut information transfer pathway, the contribution of different EEG frequency bands varies across sleep stages (**Figure 5A**). During LS, the beta band show the strong contribution; during DS, the delta band becomes the dominant contributor; during REM sleep, the alpha band exhibits the strongest contribution. At the intestinal receiving end (**Figure 5C**), the bowel sound BS2 band shows stronger statistical sensitivity during LS and REM, while the BS4 band shows higher sensitivity during DS.

**Figure 5:**
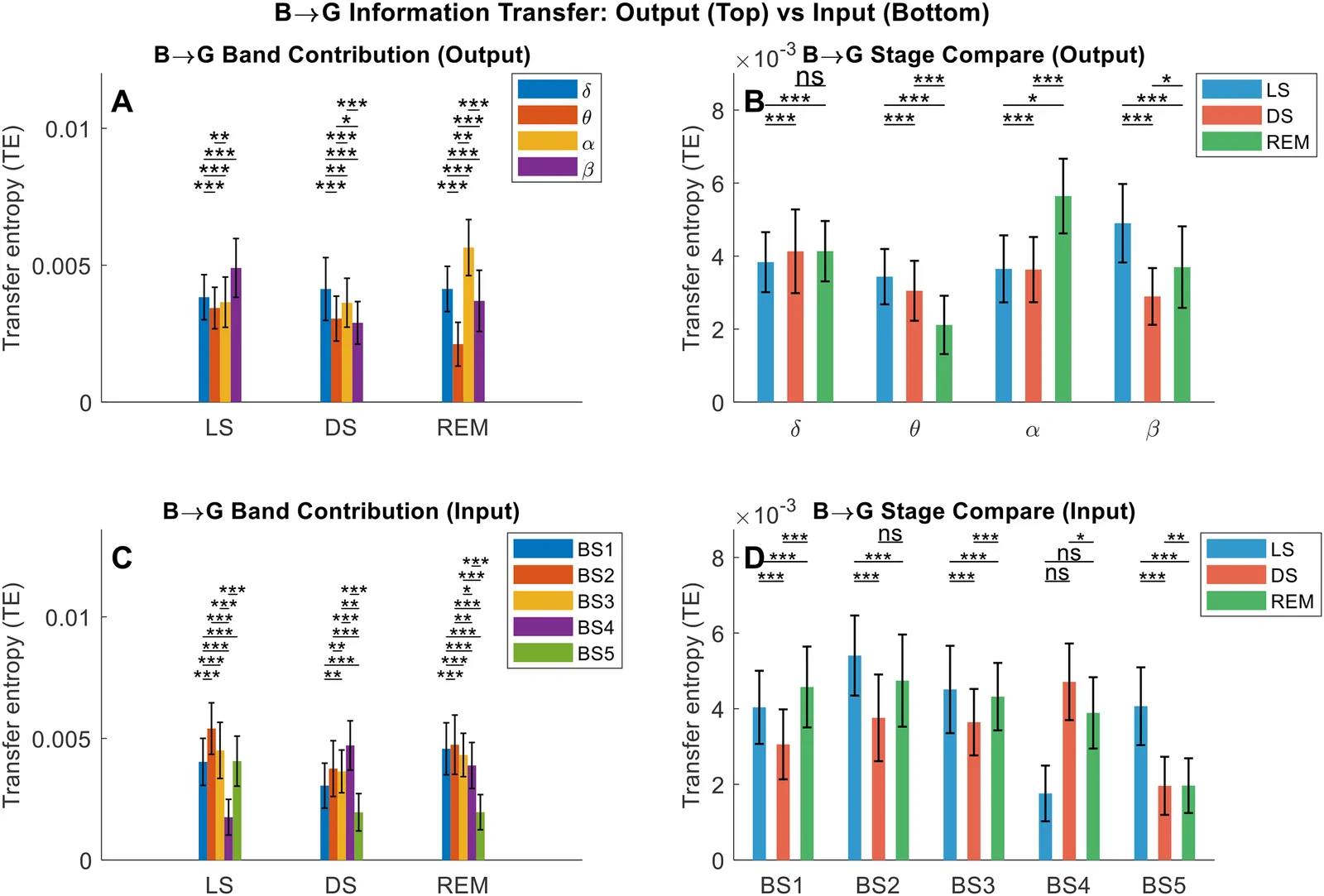
Changes in the strength of input and output information flow across different frequency bands in the brain-to-gut direction as a function of sleep stage. **(A)** Brain-to-gut output band contribution, **(B)** brain-to-gut output stage comparison, **(C)** brain-to-gut input band contribution, and **(D)** brain-to-gut input stage comparison. ns *P* > 0.05, *P < 0.05, **P < 0.01, ***P < 0.001. Values are window-level transfer entropy (mean ± SE across windows), matching the unit of analysis of the linear mixed model.

**Figure 5** also presents the changes in transfer strength of each frequency band in the brain-to-gut pathway across sleep stages (**Figures 5B, D**). The results indicate that sleep stage significantly modulates the information transfer strength of each band: in the outgoing direction, the output information of the theta, alpha and beta bands decreases during DS, while that of the delta band increases; correspondingly, in the incoming direction, the input information of the intestinal BS1, BS2, BS3, and BS5 bands decreases during DS, whereas the change in the BS4 band is not statistically significant.

In the heart-to-gut information transfer pathway, the statistical association between ANS and gut envelopes also exhibits complex band interactions and sleep-dependent characteristics. As shown in **Figure 6A**, during both LS and DS, the HF band serves as the predominant outgoing band; however, during REM sleep, the outgoing strength of the LF band is significantly higher than that of the HF band. At the intestinal receiving end (**Figure 6C**), the BS5 band acts as the core incoming band during DS, the BS2 band serves as the core incoming band during REM, while during LS, the BS2, BS3, and BS4 bands all show relatively high incoming information flow strengths. The transfer strength of the heart-to-gut pathway is also significantly modulated by sleep stage (**Figures 6B, D**). As sleep deepens, the outgoing information strengths of both the LF and HF bands in the RRI sequence gradually decrease. On the intestinal incoming side, the incoming information of the bowel sound BS1, BS2, BS3, and BS4 bands also weakens with deepening sleep.

**Figure 6:**
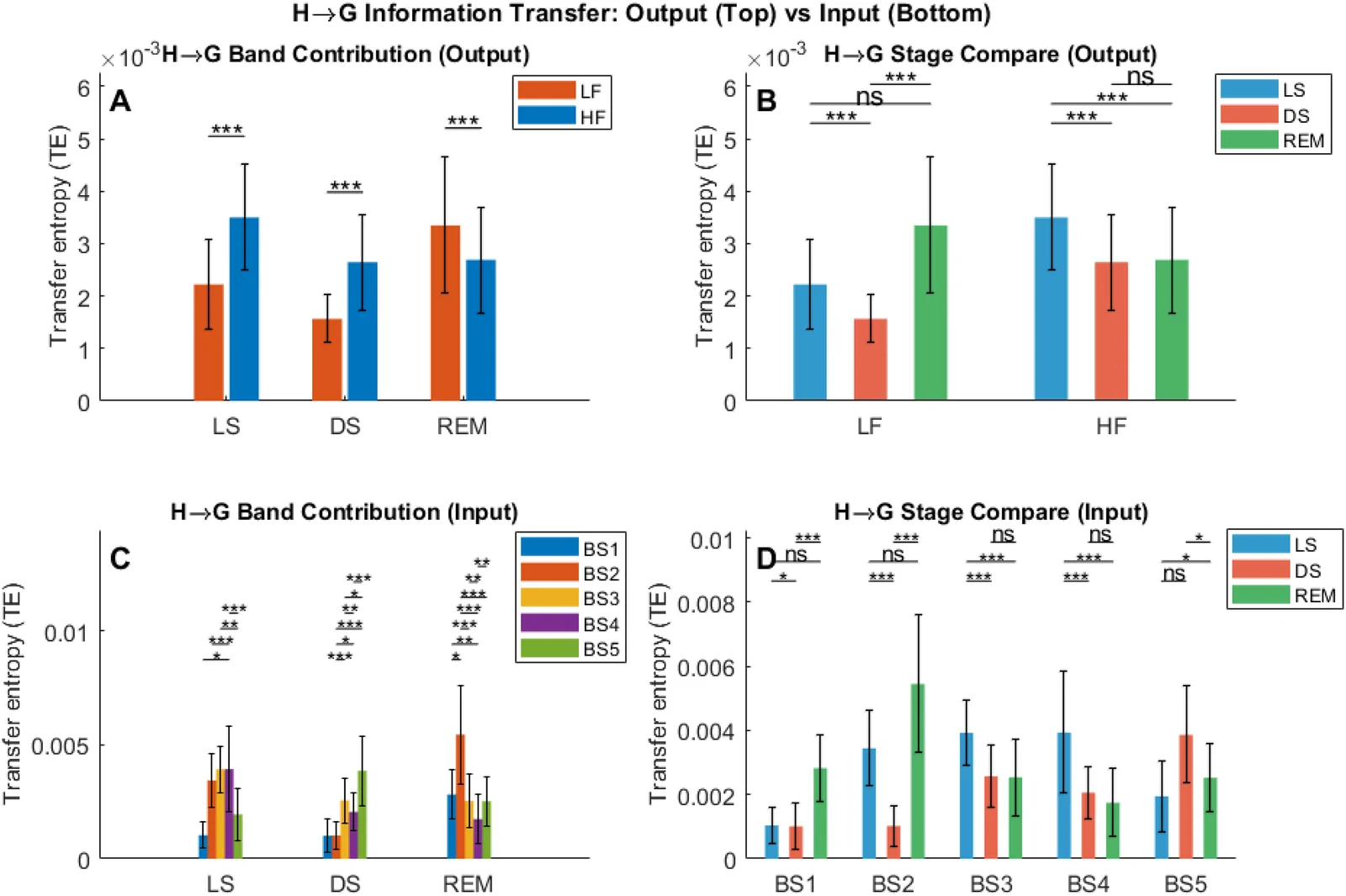
Changes in the strength of input and output information flow across different frequency bands in the heart-to-gut direction as a function of sleep stage. **(A)** Heart-to-gut output band contribution, **(B)** heart-to-gut output stage comparison, **(C)** heart-to-gut input band contribution, and **(D)** heart-to-gut input stage comparison. ns *P* > 0.05, *P < 0.05, **P < 0.01, ***P < 0.001. Values are window-level transfer entropy (mean ± SE across windows), matching the unit of analysis of the linear mixed model.

## 4 Discussion

This study employed the MuTE method to systematically delineate the dynamic, bidirectional information flow network among the brain, heart, and gut systems across the entire night of sleep for the first time. The main findings include: ①The brain-heart-gut network exhibits a hierarchical information flow structure, with the information transfer strengths in the heart-to-brain, brain-to-gut, and heart-to-gut directions being significantly higher than those in their respective reverse pathways; ②Sleep-stage dynamics differ between within-system and inter-system information flow: within-system flow (brain and gut) increases from LS to DS and weakens during REM, whereas brain-to-gut and heart-to-gut decrease during DS—yet with distinct recovery patterns during REM; ③Frequency bands within each system exhibit dynamically reorganized source–receiver roles across sleep stages, and frequency-resolved inter-system pathway analyses reveal stage- and pathway-specific contributions that are exploratory in nature; ④ The presence of a significant heart-to-gut information flow suggests the existence of a heart-gut communication pathway that may operate, at least in part, independently of higher central nervous system centers. This is anatomically supported by both sympathetic and parasympathetic innervation of the digestive tract. However, the specific contributions of vagal versus sympathetic pathways cannot be conclusively determined from HRV spectral components alone. These findings are discussed below in the context of existing literature.

### 4.1 Directional asymmetry in relation to prior literature

This study found that the information transfer strengths in the heart-to-brain, brain-to-gut, and heart-to-gut directions consistently higher than those in the corresponding reverse directions, a pattern that remains stable across different sleep stages. The dominant direction of heart-to-brain is highly consistent with previous studies: Yang et al. (Yang et al., 2021) reported that information flow from the cardiopulmonary system to the brain is stronger than the reverse direction across all sleep stages; Faes et al. (Faes et al., 2015) and Zhu et al (Zhu et al., 2025) similarly confirmed that information flow in the brain-heart network is predominantly directed from HRV to EEG. This supports the theory of somatic afferent input from the heart to the brain (Morchiladze et al., 2018), i.e., during sleep, cortical neurons shift from processing external information to processing visceral internal information. While our findings at the envelope level are consistent with this theoretical framework, it is important to emphasize that our MuTE analysis quantifies statistical prediction at the slow power envelope scale; the actual neural spike-based or oscillatory phase-coded information transfer remains to be investigated with higher temporal resolution methods. The predominance of the brain-to-gut direction is consistent with, but does not confirm, the classical top-down regulation hypothesis of the brain-gut axis (Cryan et al., 2019): the central nervous system regulates intestinal motility, secretion, and barrier function via autonomic pathways, especially the vagus nerve (Dong and Mayer, 2024; Toledo et al., 2025). This study provides information-flow evidence that the brain exhibits a leading statistical relationship with the gut even during sleep. The statistically robust heart-to-gut information flow observed in our study is consistent with the existence of peripheral autonomic pathway that modulate gut function partly through mechanisms that do not necessarily require higher central nervous system processing at the time scale of our analysis (Sun et al., 2019; Dong and Mayer, 2024). Both sympathetic and parasympathetic efferents innervate the digestive tract and could conceivably mediate such information transfer. One possible interpretation is that, in some patients, intestinal symptoms might arise from direct autonomic influences on the gut rather than from central-level dysfunction; this in turn raises the possibility that interventions targeting the autonomic nervous system may influence gut function via peripheral pathways. However, this remains a speculative hypothesis derived from physiological observations in healthy young adults, and the present data do not establish a causal link. It therefore requires direct testing in patient populations before any clinical conclusions can be drawn.

### 4.2 Sleep stage-dependent patterns of information flow: dissociation between within-system and inter-system dynamics

This study reveals a fundamental dissociation between within-system and inter-system information flow dynamics across sleep stages (**Figure 2**). Within-system information transfer strengths—particularly within the brain and gut—gradually increase from LS to DS and weaken during REM sleep (**Figures 2A, C**), indicating that deep sleep serves as a critical window for enhanced internal information exchange at the envelope level within each system. This enhancement in internal information flow is accompanied by dynamically reorganized statistical source–receiver relationships among frequency bands within each system (detailed in **Figure 3** and discussed in the following section), consistent with the established view that deep sleep facilitates intracortical synchronization and autonomic regulation (Spencer and Hu, 2020). This enhanced multisystem synchronization may contribute to the neurophysiological processes underlying the restorative functions of deep sleep. In striking contrast, inter-system information flow exhibits a different pattern (**Figures 2D–F**). Heart-to-gut and brain-to-gut pathways show significantly decreased information transfer during DS compared to LS, whereas heart-to-brain information transfer increases progressively from LS to DS and further to REM. This suppression of cross-system envelope-level interactions during deep sleep may reflect a state of reduced inter-organ communication that facilitates the restorative functions of slow-wave sleep. However, the recovery during REM sleep differentiates these pathways: the heart-to-brain and heart-to-gut pathways show a significant rebound during REM sleep, consistent with the reopening of the cortex to peripheral input due to desynchronization during REM sleep (Faes et al., 2015; Yang et al., 2021) and the persistence of autonomic influences on the gut. In contrast, the brain-to-gut pathway does not exhibit a significant rebound during REM sleep, indicating that the brain-to-gut direction of association remains attenuated even when cortical activity becomes desynchronized. This differential pattern—progressively increasing dynamics for heart-to-brain, U-shaped dynamics for heart-to-gut, and L-shaped dynamics for the descending pathway (brain to gut)—implies that sleep stages exert dissociable modulatory effects on afferent/autonomic versus efferent directions: ascending and peripheral autonomic pathways are more sensitive to cortical state changes and recover during REM, whereas the descending cortico-visceral pathway may be subjected to a more persistent suppression that extends into REM sleep. This finding provides new information-theoretic evidence for the asymmetric modulation of ascending and descending pathways during sleep, complementing previous neurophysiological studies (Coenen, 2024). However, our information-theoretic analysis alone cannot identify the specific anatomical structures or neural circuits responsible for this differential modulation. Future studies combining targeted neuromodulation or lesion experiments are needed to validate these pathway-specific dynamics and to elucidate their underlying neural substrates.

### 4.3 Frequency-specific directed association: exploratory observations

At the frequency-band level, each system exhibits dynamically reorganized statistical source–receiver roles across sleep stages, and the dominant contributing frequency bands in each inter-system pathway shift depending on sleep stage (detailed in **Figures 3**-**6**). These frequency-resolved findings indicate that information transfer at the envelope level involves complex, stage-dependent, and pathway-specific frequency interactions. However, unlike the macroscopic hierarchical structure and sleep-stage dynamics discussed above, the specific physiological interpretations of these band-level directional patterns remain unclear due to the absence of directly comparable literature. Existing studies have largely focused on correlational relationships between spectral powers rather than directional information flow at the frequency-band level (Miyashita et al., 2003; Faes et al., 2015; Yang et al., 2021), and the use of bowel sound frequency bands as information-theoretic nodes is itself a recent methodological development with limited prior validation (Wang et al., 2022a, 2023). Moreover, the field of brain–heart interaction research has predominantly examined bidirectional coupling between the brain and the heart within a bivariate framework, largely without considering the gut as an interacting node (Faes et al., 2015; Yang et al., 2021; Zhu et al., 2025). In contrast, the present study employed a multivariate transfer entropy approach that explicitly conditions on gut activity when quantifying heart-to-brain and brain-to-heart information flow. This methodological distinction is non-trivial: because the gut receives both central and autonomic inputs and, in turn, generates afferent signals that may modulate autonomic outflow, the inclusion of gut dynamics as a conditioning variable could alter the estimated brain–heart coupling patterns compared to conventional bivariate analyses. In other words, what appears as a direct brain–heart interaction in a bivariate framework may partly reflect indirect influences mediated by, or shared with, gut-related autonomic processes. The comprehensive investigation of brain–heart–gut interactions across all three systems remains scarce, and the extent to which gut activity modulates or confounds brain–heart coupling during sleep has not been systematically characterized. Therefore, the present findings—particularly those involving frequency-resolved interactions—should be interpreted as exploratory statistical associations that require independent validation through targeted experiments.

### 4.4 Methodological considerations and alternative interpretations

Several considerations regarding the interpretation of bowel sound signals warrant attention. First, bowel sounds are indirect acoustic surrogates of gastrointestinal motility rather than direct measures of intestinal muscle contraction or enteric neural activity, and the correspondence between specific frequency bands and distinct contractile patterns—such as propulsive peristalsis versus segmental contractions—remains to be established. Second, bowel sound recordings are susceptible to multiple physiological and technical confounders, including intra-abdominal pressure, body position, sensor placement, tissue attenuation, and ambient noise, all of which may introduce variability into power spectral estimates. However, our within-subject, sleep-stage comparative design mitigates the impact of time-invariant confounders (e.g., sensor characteristics, abdominal wall transmission, and sensor placement), as these factors remain stable throughout a single overnight recording. With these considerations in mind, our findings should be viewed as statistical evidence for frequency-specific information flow involving bowel sound envelopes, rather than as definitive identification of specific motility patterns or direct neural pathways.

It is worth noting that a more parsimonious alternative interpretation exists for the observed hierarchical directional patterns: global state factors such as sleep stage, respiration, and body movements may simultaneously modulate all three systems, while differences in filtering properties and response latencies across modalities could produce statistical appearances resembling a hierarchy. Therefore, our results should be interpreted cautiously as exploratory statistical associations derived from information-theoretic measures, rather than as strict organ-to-organ causal chains. Future neuromodulation or pharmacological-blockade experiments will be needed to distinguish genuine information transfer from common-driver effects. Additionally, the use of relative band powers introduces compositional closure constraints within each physiological system, as bands share a common denominator and are not independent, which may artificially influence within-system MuTE estimates. Importantly, this concern does not extend to the between-system directional findings, which were fully replicated when absolute band powers were used (**Supplementary File 2**). However, within-system band-level role assignments showed some sensitivity to this normalization and should therefore be interpreted as exploratory. Beyond this methodological consideration, a critical methodological consideration pertains to the temporal resolution of our analysis. As described in the Methods, all physiological signals were converted into 1-Hz power time series via sliding-window spectral estimation. This operation effectively extracts the slow power envelope of each signal while discarding the fast oscillatory dynamics (e.g., precise phase information, transient spike-like events) present in the original carrier waveforms. Consequently, the directed information flow quantified by MuTE in this study reflects statistical predictive relationships among slow amplitude co-modulations, rather than information transfer at the level of the original carrier oscillations. Our findings should therefore be interpreted as evidence of macro-scale regulatory interactions at the timescale of seconds to minutes, rather than fast neural coding or spike-based information transfer. A related methodological limitation concerns the uniform maximum time delay (μ = 10 s) used for candidate matrix construction. While the subsequent non-uniform embedding step mitigates this issue to some extent by selecting statistically optimal lags for each node, the search range is still confined within 10 s. This inherently biases the analysis toward faster cardiorespiratory and brain dynamics, while slower gastrointestinal responses (e.g., motility cycles > 10 s) may be undersampled or missed entirely. Future studies should consider physiologically guided, system-specific embedding ranges to better capture the full spectrum of multi-organ interactions. An additional methodological consideration concerns the use of a single EEG channel (O2) to represent cortical activity. While this choice was justified by dimensionality reduction, signal stability, and prior brain-heart coupling studies, we acknowledge that frontal and central electrodes—which are more directly implicated in sleep regulation and autonomic control—may yield distinct coupling patterns. It is therefore plausible that information flow involving frontal or central EEG would differ from that observed with occipital EEG, particularly in autonomic pathways (e.g., heart-to-brain or brain-to-gut). Future studies incorporating multi-lead EEG recordings are needed to characterize the spatial topography of brain-heart-gut information flow and to determine whether the present findings generalize across cortical regions. Another methodological consideration pertains to stationarity and sleep-stage boundaries. In the present data, no window combines epochs from different stages. Within each stage, however, the power time series were concatenated in chronological order to obtain sufficient sample sizes, which necessarily joins temporally non-contiguous segments of the same stage. On the one hand, sleep staging itself discretizes an inherently continuous neurophysiological process, and signals within a single stage are conventionally treated as approximately stationary over timescales of minutes, such that stage-specific windows exclude the dominant source of nonstationarity. On the other hand, the absence of formal stationarity screening and micro-arousal exclusion leaves open the possibility that residual transient activations or splice-point artifacts may concurrently modulate all three modalities and thereby inflate apparent coupling. These are intrinsic limitations of the present envelope-level, stage-specific design, and future studies should address them through explicit stationarity criteria, micro-arousal annotation, and uninterrupted within-stage segments.

### 4.5 Limitations and future directions

Beyond these considerations, this study has some limitations. First, this study focused on healthy young adults and validated the feasibility of the brain-heart-gut interaction analysis framework. However, the generalizability of the findings needs to be further verified in different age groups and in patients with sleep disorders or functional gastrointestinal disorders. Second, the directed associations reported in this study are inferred on the basis of statistical information theory; while the directional predominance observed is consistent with known anatomical pathways (e.g., vagal efferents, brain-gut descending projections), MuTE does not constitute proof of biological causality. Future studies could combine neuromodulation techniques such as vagus nerve stimulation, pharmacological blockade, or closed-loop transcranial electrical stimulation to target specific pathways (e.g., the heart-gut HF pathway, the brain-gut δ pathway) for validation. Third, the interpretation of HRV spectral components as proxies of autonomic activity requires caution. The LF band reflects mixed sympathetic and parasympathetic influences rather than pure sympathetic tone, and the HF band is substantially modulated by respiration. Although we recorded respiratory signals via PSG, we did not perform explicit respiratory correction in the main analysis due to the natural fluctuations of respiration during sleep. Therefore, we refer to LF and HF as HRV spectral measures and interpret their information transfer patterns as spectral associations rather than direct neural pathway confirmations. Future studies incorporating concurrent respiratory correction or pharmacological autonomic blockade would be valuable to further validate the neural specificity of these pathways. Fourth, higher temporal resolution analyses (e.g., phase-amplitude coupling, cross-frequency directionality, or event-based analysis of transient bursts) would complement the present envelope-level findings by probing faster information transfer mechanisms. Notably, the control analyses using time-shifted surrogates and sleep-stage conditioning suggested that our envelope-level TE estimates reflect genuine co-modulation patterns beyond mere autocorrelation or state-driven artifacts, which supports the robustness of our macro-scale findings. Fifth, the bowel sound frequency band boundaries used in this study are empirically derived from the literature rather than validated against independent physiological measurements. The acoustic spectrum of bowel sounds is influenced by multiple technical and anatomical factors, and there is currently no established consensus on standardized frequency band definitions for bowel sound analysis. Therefore, our frequency band-specific findings should be interpreted as exploratory spectral associations rather than definitive physiological pathway identifications. Finally, the influences of factors such as sex and circadian rhythms on brain-heart-gut information flow merit further investigation.

## 5 Conclusion

This study achieved non-invasive dynamic assessment of directional information flow at the level of slow power envelope co-modulations among the brain, heart, and gut systems during sleep by synchronously collecting EEG, ECG, and bowel sound signals and using the multivariate transfer entropy method. Theoretically, this study suggests that differential patterns of information flow in ascending versus descending pathways across sleep stages, providing a new information-theoretic perspective for understanding the integrated physiological regulation during sleep at the macro-scale. Clinically, these findings are exploratory and hypothesis-generating. Whether the observed slow power-envelope associations reflect anatomical pathways, causal regulation, or clinically relevant biomarkers cannot be determined from the present data and requires validation in independent and clinical populations.

## Data Availability

The raw data supporting the conclusions of this article will be made available by the authors, without undue reservation.

## References

[B1] Ali M. K. Chen J. D. Z. (2023). Roles of heart rate variability in assessing autonomic nervous system in functional gastrointestinal disorders: a systematic review. Diagnostics (Basel) 13, 293. doi: 10.3390/diagnostics13020293 36673103 PMC9857852

[B2] Ali M. K. Liu L. Chen J.-H. Huizinga J. D. (2021). Optimizing autonomic function analysis via heart rate variability associated with motor activity of the human colon. Front. Physiol. 12, 619722. doi: 10.3389/fphys.2021.619722 34267670 PMC8275990

[B3] Al-Naggar N. Q. Al-Udyni M. H. (2018). Performance of adaptive noise cancellation with normalized last-mean-square based on the signal-to-noise ratio of lung and heart sound separation. J. Healthc Eng. 2018, 9732762. doi: 10.1155/2018/9732762 30123445 PMC6079454

[B4] Berry R. B. Brooks R. Gamaldo C. Harding S. M. Lloyd R. M. Quan S. F. . (2017). AASM scoring manual updates for 2017 (Version 2.4). J. Clin. Sleep Med. 13, 665–666. doi: 10.5664/jcsm.6576 28416048 PMC5406946

[B5] Chen X. Xu X. Liu A. McKeown M. Wang Z. (2017). The use of multivariate EMD and CCA for denoising muscle artifacts from few-channel EEG recordings. IEEE Trans. Instrum. Meas. PP, 1–12. doi: 10.1109/TIM.2017.275939825079929

[B6] Cheng W. Chen H. Tian L. Ma Z. Cui X. (2023). Heart rate variability in different sleep stages is associated with metabolic function and glycemic control in type 2 diabetes mellitus. Front. Physiol. 14, 1157270. doi: 10.3389/fphys.2023.1157270 37123273 PMC10140569

[B7] Coenen A. (2024). Sensory gating and gaining in sleep: the balance between the protection of sleep and the safeness of life (a review). J. Sleep Res. 33, e14152. doi: 10.1111/jsr.14152 38286435

[B8] Cryan J. F. O’Riordan K. J. Cowan C. S. M. Sandhu K. V. Bastiaanssen T. F. S. Boehme M. . (2019). The microbiota-gut-brain axis. Physiol. Rev. 99, 1877–2013. doi: 10.1152/physrev.00018.2018 31460832

[B9] Dong T. S. Mayer E. (2024). Advances in brain-gut-microbiome interactions: a comprehensive update on signaling mechanisms, disorders, and therapeutic implications. Cell. Mol. Gastroenterol. Hepatol. 18, 1–13. doi: 10.1016/j.jcmgh.2024.01.024 38336171 PMC11126987

[B10] Ekhlasi A. Nasrabadi A. M. Mohammadi M. (2023). Improving transfer entropy and partial transfer entropy for relative detection of effective connectivity strength between time series. Commun. Nonlinear Sci. Numer. Simul. 126, 107449. doi: 10.1016/j.cnsns.2023.10744942574925

[B11] Faes L. Marinazzo D. Jurysta F. Nollo G. (2015). Linear and non-linear brain-heart and brain-brain interactions during sleep. Physiol. Meas. 36, 683–698. doi: 10.1088/0967-3334/36/4/683 25799205

[B12] Ficek J. Radzikowski K. Nowak J. K. Yoshie O. Walkowiak J. Nowak R. (2021). Analysis of gastrointestinal acoustic activity using deep neural networks. Sensors 21, 7602. doi: 10.3390/s21227602 34833679 PMC8618847

[B13] Geng W. Cao X. Liao W. Yang Y. (2026). Clinical applications and mathematical models of bowel bounds. Biomedicines 14, 581. doi: 10.3390/biomedicines14030581 41898228 PMC13023620

[B14] Goh N. Lim F. Kwan J. M. C. Widarsa J. Agrawal K. Ko H. . (2025). Acoustic sensing and analysis of bowel sounds in irritable bowel syndrome - recent engineering developments and clinical applications. Sensors Actuators A. Phys. 394, 116910. doi: 10.1016/j.sna.2025.11691042574925

[B15] Granger C. W. J. (1969). Investigating causal relations by econometric models and cross-spectral methods. Econometrica 37, 424–438. doi: 10.2307/1912791

[B16] Harmah D. J. Li C. Li F. Liao Y. Wang J. Ayedh W. M. A. . (2019). Measuring the non-linear directed information flow in schizophrenia by multivariate transfer entropy. Front. Comput. Neurosci. 13, 85. doi: 10.3389/fncom.2019.00085 31998105 PMC6966771

[B17] Ivanov P. C. (2021). The new field of network physiology: building the human physiolome. Front. Netw. Physiol. 1, 711778. doi: 10.3389/fnetp.2021.711778 36925582 PMC10013018

[B18] Jeanne R. Piton T. Minjoz S. Bassan N. Le Chenechal M. Semblat A. . (2023). Gut-brain coupling and multilevel physiological response to biofeedback relaxation after a stressful task under virtual reality immersion: a pilot study. Appl. Psychophysiol. Biofeedback 48, 109–125. doi: 10.1007/s10484-022-09566-y 36336770

[B19] Jorgensen G. Downey C. Goldin J. Melehan K. Rochford P. Ruehland W. (2020). An Australasian commentary on the AASM manual for the scoring of sleep and associated events. Sleep Biol. Rhythms 18, 163–185. doi: 10.1007/s41105-020-00259-930311153

[B20] Koizumi K. Hamaguchi T. Tayama J. Fukudo S. (2022). A method of generating a classifier that determines the presence or absence of IBS symptoms by supervised learning from the frequency analysis of electroencephalogram data. Front. Biosci. (Landmark Ed) 27, 187. doi: 10.31083/j.fbl2706187 35748263

[B21] Kraskov A. Stögbauer H. Grassberger P. (2004). Estimating mutual information. Phys. Rev. E 69, 66138. doi: 10.1103/PhysRevE.69.066138 15244698

[B22] Kuo T. B. J. Chen C. Hsu Y. Yang C. C. H. (2016). EEG beta power and heart rate variability describe the association between cortical and autonomic arousals across sleep. Auton. Neurosci. 194, 32–37. doi: 10.1016/j.autneu.2015.12.001 26681575

[B23] Lyu J. Shi W. Zhang C. Yeh C.-H. (2023). A novel sleep staging method based on EEG and ECG multimodal features combination. IEEE Trans. Neural Syst. Rehabil. Eng. 31, 4073–4084. doi: 10.1109/TNSRE.2023.3323892 37819827

[B24] Miyashita T. Ogawa K. Itoh H. Arai Y. Ashidagawa M. Uchiyama M. . (2003). Spectral analyses of electroencephalography and heart rate variability during sleep in normal subjects. Auton. Neurosci. 103, 114–120. doi: 10.1016/s1566-0702(02)00259-x 12531405

[B25] Montalto A. Faes L. Marinazzo D. (2014). MuTE: a MATLAB toolbox to compare established and novel estimators of the multivariate transfer entropy. PloS One 9, e109462. doi: 10.1371/journal.pone.0109462 25314003 PMC4196918

[B26] Morchiladze M. M. Silagadze T. K. Silagadze Z. K. (2018). Visceral theory of sleep and origins of mental disorders. Med. Hypotheses 120, 22–27. doi: 10.1016/j.mehy.2018.07.023 30220335

[B27] Mróz M. Czub M. Brytek-Matera A. (2022). Heart rate variability-an index of the efficacy of complementary therapies in irritable bowel syndrome: a systematic review. Nutrients 14, 3447. doi: 10.3390/nu14163447 36014953 PMC9416471

[B28] Orr W. C. Crowell M. D. Lin B. Harnish M. J. Chen J. D. Z. (1997). Sleep and gastric function in irritable bowel syndrome: derailing the brain-gut axis. Gut 41, 390–393. doi: 10.1136/gut.41.3.390 9378397 PMC1891498

[B29] Pan D. Li J. Chen S. Gu S. Jiang M. Xu Q. (2025). Microbiota-gut-brain axis pathogenesis and targeted therapeutics in sleep disorders. Front. Neurol. 16, 1721606. doi: 10.3389/fneur.2025.1721606 41458114 PMC12740866

[B30] Pilia N. Nagel C. Lenis G. Becker S. Dössel O. Loewe A. (2021). ECGdeli - an open source ECG delineation toolbox for MATLAB. SoftwareX 13, 100639. doi: 10.1016/j.softx.2020.10063942574925

[B31] Platiša M. M. Radovanović N. N. Pernice R. Barà C. Pavlović S. U. Faes L. (2023). Information-theoretic analysis of cardio-respiratory interactions in heart failure patients: effects of arrhythmias and cardiac resynchronization therapy. Entropy (Basel) 25, 1072–1086. doi: 10.3390/e25071072 37510019 PMC10378632

[B32] Ramlee F. Sanborn A. N. Tang N. K. Y. (2017). What sways people’s judgment of sleep quality? A quantitative choice-making study with good and poor sleepers. Sleep 40, zsx091. doi: 10.1093/sleep/zsx091 28525617 PMC5804994

[B33] Richards P. Thornberry N. A. Pinto S. (2021). The gut–brain axis: identifying new therapeutic approaches for type 2 diabetes, obesity, and related disorders. Mol. Metab. 46, 101175. doi: 10.1016/j.molmet.2021.101175 33548501 PMC8085592

[B34] Rodenbeck A. Danker-Hopfe H. Geisler P. Binder R. Lund R. Raschke F. . (2015). Ergänzende regeln zu frequenzen und graphoelementen der schlafstadienanalyse. Somnologie 19, 51–60. doi: 10.1007/s11818-015-0696-y30311153

[B35] Schoch S. F. Castro-Mejía J. L. Krych L. Leng B. Kot W. Kohler M. . (2022). From alpha diversity to Zzz: interactions among sleep, the brain, and gut microbiota in the first year of life. Prog. Neurobiol. 209, 102208. doi: 10.1016/j.pneurobio.2021.102208 34923049

[B36] Schreiber T. (2000). Measuring information transfer. Phys. Rev. Lett. 85, 461–464. doi: 10.1103/PhysRevLett.85.461 10991308

[B37] Sclocco R. Fisher H. Staley R. Han K. Mendez A. Bolender A. . (2022). Cine gastric MRI reveals altered gut-brain axis in functional dyspepsia: gastric motility is linked with brainstem-cortical fMRI connectivity. Neurogastroenterol. Motil. 34, e14396. doi: 10.1111/nmo.14396 35560690 PMC9529794

[B38] Sen P. Molinero-Perez A. O’Riordan K. J. McCafferty C. P. O’Halloran K. D. Cryan J. F. (2021). Microbiota and sleep: awakening the gut feeling. Trends Mol. Med. 27, 935–945. doi: 10.1016/j.molmed.2021.07.004 34364787

[B39] Shi X. Hu Y. Zhang B. Li W. Chen J. D. Liu F. (2021). Ameliorating effects and mechanisms of transcutaneous auricular vagal nerve stimulation on abdominal pain and constipation. JCI Insight 6, e150052. doi: 10.1172/jci.insight.150052 34138761 PMC8410029

[B40] Sidhik S. (2015). Comparative study of Birge–Massart strategy and unimodal thresholding for image compression using wavelet transform. Optik 126, 5952–5955. doi: 10.1016/j.ijleo.2015.08.12742574925

[B41] Sood D. Dadwal S. Jain S. Mazhar I. J. Goyal B. Garapati C. . (2026). Prospective of colorectal cancer screening, diagnosis, and treatment management using bowel sounds leveraging artificial intelligence. Cancers 18, 340. doi: 10.3390/cancers18020340 41595259 PMC12838979

[B42] Spencer N. J. Hu H. (2020). Enteric nervous system: sensory transduction, neural circuits and gastrointestinal motility. Nat. Rev. Gastroenterol. Hepatol. 17, 338–351. doi: 10.1038/s41575-020-0271-2 32152479 PMC7474470

[B43] Sun Y. Li L. Xie R. Wang B. Jiang K. Cao H. (2019). Stress triggers flare of inflammatory bowel disease in children and adults. Front. Pediatr. 7, 432. doi: 10.3389/fped.2019.00432 31709203 PMC6821654

[B44] Tian Z. K. Chen K. Li S. McLaughlin D. W. Zhou D. (2024). Causal connectivity measures for pulse-output network reconstruction: analysis and applications. Proc. Natl. Acad. Sci. U.S.A. 121, e2305297121. doi: 10.1073/pnas.2305297121 38551842 PMC10998614

[B45] Toledo M. Martínez-Martínez S. Van Hul M. Laudo B. Eyre E. Pelicaen R. . (2025). Rapid modulation of gut microbiota composition by hypothalamic circuits in mice. Nat. Metab. 7, 1123–1135. doi: 10.1038/s42255-025-01280-3 40263603 PMC12198007

[B46] Valenza G. Greco A. Gentili C. Lanata A. Sebastiani L. Menicucci D. . (2016). Combining electroencephalographic activity and instantaneous heart rate for assessing brain-heart dynamics during visual emotional elicitation in healthy subjects. Philos. Trans. A. Math. Phys. Eng. Sci. 374, 20150176. doi: 10.1098/rsta.2015.0176 27044990 PMC4822439

[B47] Vlachos I. Kugiumtzis D. (2010). Nonuniform state-space reconstruction and coupling detection. Phys. Rev. E 82, 16207. doi: 10.1103/PhysRevE.82.016207 20866707

[B51] Wang Y. Chen C. Chen W. (2022c). Nonlinear directed information flow estimation for fNIRS brain network analysis based on the modified multivariate transfer entropy. Biomed. Signal Process. Control 74, 103422. doi: 10.1016/j.bspc.2021.10342242574925

[B48] Wang G. Chen Y. Liu H. Yu X. Han Y. Wang W. . (2023). Differences in intestinal motility during different sleep stages based on long-term bowel sounds. Biomed. Eng. Online 22, 105. doi: 10.1186/s12938-023-01166-z 37919731 PMC10623717

[B52] Wang Z. Wang Z. Lu T. Chen W. Yan W. Yuan K. . (2022d). The microbiota-gut-brain axis in sleep disorders. Sleep Med. Rev. 65, 101691. doi: 10.1016/j.smrv.2022.101691 36099873

[B49] Wang G. Yang Y. Chen S. Fu J. Wu D. Yang A. . (2022a). Flexible dual-channel digital auscultation patch with active noise reduction for bowel sound monitoring and application. IEEE J. Biomed. Health Inform. 26, 2951–2962. doi: 10.1109/JBHI.2022.3151927 35171784

[B50] Wang T. Yang J. Song Y. Pang F. Guo X. Luo Y. (2022b). Interactions of central and autonomic nervous systems in patients with sleep apnea-hypopnea syndrome during sleep. Sleep Breath 26, 621–631. doi: 10.1007/s11325-021-02429-6 34231085

[B53] Wang Z. Yuan K. Ji Y. Li S. Shi L. Wang Z. . (2022e). Alterations of the gut microbiota in response to total sleep deprivation and recovery sleep in rats. Nat. Sci. Sleep 14, 121–133. doi: 10.2147/NSS.S334985 35115853 PMC8800865

[B54] Wiener N. (1956). “ The theory of prediction,” in Modern mathematics for engineers. Beckenbach, New York: McGraw-Hill.

[B55] Xi X. Wu X. Zhao Y.-B. Wang J. Kong W. Luo Z. (2021). Cortico-muscular functional network: an exploration of cortico-muscular coupling in hand movements. J. Neural Eng. 18, 046084. doi: 10.1088/1741-2552/ac0586 34038874

[B56] Yang J. Pan Y. Wang T. Zhang X. Wen J. Luo Y. (2021). Sleep-dependent directional interactions of the central nervous system-cardiorespiratory network. IEEE Trans. Biomed. Eng. 68, 639–649. doi: 10.1109/TBME.2020.3009950 32746063

[B57] Zhao D. Zou B. Do Q. L. Wu S.-K. Shen Y. Yang Y. . (2026). Circadian rhythms and gut microbiota dysbiosis: emerging gut-brain axis pathways in insomnia pathophysiology and therapeutics. Brain Behavior Immun. 132, 106203. doi: 10.1016/j.bbi.2025.106203 41330454

[B58] Zhu S. Sun W. Xie M. Tong Z. Wang J. (2025). Amplitude-aware symbolic transfer entropy for heart-brain coupling analysis across sleep stages. AIP Adv. 15, 035149. doi: 10.1063/5.024515342469603

