# Supplementary material for: Slow power-envelope associations among brain, heart, and gut signals during sleep: an exploratory multivariate transfer entropy study
Source: Front Physiol. 2026 Oct 20;17:1924709. doi: 10.3389/fphys.2026.1924709 (PMC13727474; doi:10.3389/fphys.2026.1924709)
Supplement: Supplementary file 1 [file SupplementaryFile1.docx]

**Supplementary Figure S1. Participant flow diagram.**
