# Supplementary material for: Slow power-envelope associations among brain, heart, and gut signals during sleep: an exploratory multivariate transfer entropy study
Source: Front Physiol. 2026 Oct 20;17:1924709. doi: 10.3389/fphys.2026.1924709 (PMC13727474; doi:10.3389/fphys.2026.1924709)
Supplement: Supplementary file 2 [file Table1.docx]

Table S1. Multiple-testing correction: families, models, and number of tests.

| Family | Research question | Model | No. tests | Sources |
| --- | --- | --- | --- | --- |
| 1 | Does TE vary across sleep stage, for each directed connection? | TE ~ Stage + (1\|Participant) (LMM F-test) | 110 | Screening |
| 2 | Directional asymmetry (Input vs. Output), system level and within-system per band | TE ~ Direction + (1\|Participant) | 42 | Fig. 1 + Fig. 3 |
| 3 | Pairwise between-band contrasts, within each system/pathway × stage | TE ~ Band + (1\|Participant) +coefTest | 102 | Fig. 4–6 |
| 4 | Pairwise between-stage contrasts, for each band/pathway | TE ~ Stage + (1\|Participant) +coefTest | 150 | Fig. 2 + Fig. 3 + Fig. 4–6 |
|  |  | Total | 404 |  |

Table S1. Multiple-testing correction structure. All p-values within a family were corrected jointly using the Benjamini–Hochberg false-discovery-rate procedure at q < 0.05; each p-value belongs to exactly one family. Families 2–4 pool all tests of the same type across figures before correction. Post-hoc pairwise stage contrasts following a significant Family-1 connection were additionally Bonferroni-corrected (multiplier = 3). LS, light sleep; DS, deep sleep; REM, rapid eye movement sleep.
